# Tryptophan modulation in individuals with attention deficit hyperactivity disorder: a systematic review

**DOI:** 10.1007/s00702-022-02478-5

**Published:** 2022-03-14

**Authors:** Larisa Maria Dinu, Nachaphol Phattharakulnij, Eleanor Jane Dommett

**Affiliations:** King’s College London Institute of Psychiatry Psychology and Neuroscience, London, UK

**Keywords:** Tryptophan, ADHD, Attention deficit hyperactivity disorder, Diet, Serotonin

## Abstract

The serotonergic system is implicated in ADHD, but the impact of serotonin’s precursor molecule, tryptophan, on ADHD symptomology remains unclear. Systematic searches of randomised controlled trials with an experimental tryptophan intervention in children and adults with ADHD identified 14 studies measuring core and related symptoms of the condition. Risk of bias was assessed using the Cochrane Risk of Bias tool. The 14 studies all used acute tryptophan depletion procedures, and most did not investigate core ADHD symptoms (inattention, impulsivity, hyperactivity) as primary outcome measures. Only two studies examined attention and revealed mixed effects of tryptophan. Similar effects were found for impulsivity in a small number of studies. No studies investigated hyperactivity. Most studies focused on reactive aggression, but samples were heterogenous and small, rendering potential meta-analyses inconclusive or misleading. However, the narrative analysis indicates tryptophan interventions may impact reactive aggression. More research is needed on the effect of tryptophan modulation on core ADHD symptoms, especially in adults, using more diverse samples to determine potential as an intervention. From current data, tryptophan modulation appears to alter aggressive behaviour in ADHD; however, the available studies were insufficient for the planned meta-analysis.

## Introduction

Attention Deficit Hyperactivity Disorder (ADHD) is the most common behavioural disorder, characterized by inattention, impulsivity and hyperactivity (American Psychiatric Association 2000). It is estimated to affect 5% of children and adolescents worldwide (Polanczyk et al. 2015; Zalsman and Shilton 2016) with around two thirds of those continuing to experience ADHD as adults (Faraone et al. 2006). In addition to those first experiencing ADHD as children, it has been suggested that the condition can develop in adults (Caye et al. 2016; Moffitt et al. 2015). Adult prevalence is estimated to be between 2 and 5% (Simon et al. 2009). Irrespective of age of onset, the condition results in significant functional impairment (National Institute for Health and Clinical Excellence 2019). Children with ADHD are at a higher risk of developing learning, behavioural and emotional problems, which contribute to their poorer educational outcomes (Faraone et al. 2000). Adults with the condition typically have a lower occupational status (Doggett 2004) and experience problems with communication and relationships—they are more likely to report problems at work and divorce (Faraone et al. 2000). Subsequently, ADHD is associated with reduced quality of life in patients and their families (Danckaerts et al. 2010).

Despite the high prevalence of ADHD and the significant functional impairment it causes, the neural basis of the condition is still poorly understood (Biederman 2005). Dysregulation of dopamine and noradrenaline has been widely implicated in the pathophysiology of the condition (Del Campo et al. 2011) with evidence from genetic studies, imaging and animal models (Faraone and Biederman 1998; Kieling et al. 2008; Russell et al. 2005). Furthermore, the most common treatment for ADHD is psychostimulant medication (e.g. methylphenidate, amphetamine) which works primarily through increasing catecholamine availability in striatal and cortical brain regions (Faraone 2018). Despite a high response rate (80%), this medication does not work for everyone and carries important side effects, including tachycardia and insomnia, coupled with concerns about the medication being abused or misused (Darredeau et al. 2007). Non-stimulant medication acting predominantly on noradrenaline (e.g. atomoxetine) can offer an alternative approach; however, the response rate is around 67%, and it continues to carry side effects, albeit less severe.

The limited efficacy of drug treatments acting on the dopaminergic and noradrenergic systems, and the gaps in our knowledge of the aetiology of ADHD, introduces the possibility that other neurotransmitter systems might be involved. One such neurotransmitter is serotonin (5-HT), which is linked to a range of cognitive and emotional functions (Moret and Briley 2011). Critically, it can be linked to the core symptoms of ADHD. Altered 5-HT functioning is associated with impulsivity (Pattij and Vanderschuren 2008). It was initially hypothesized that decreased 5-HT could result in heightened impulsivity, but the relationship appears to be more complicated than this, possibly in part due to interactions with dopamine (Oades 2008; Pattij and Vanderschuren 2008). Serotonin has also been linked to attention regulation (Koopman et al. 2016; Weinberg-Wolf et al. 2018) and the default mode network (DMN), which is thought to be altered in ADHD (Harikumar et al. 2021). Furthermore, there is  now mounting evidence for altered serotonergic genes in ADHD (Oades et al. 2008; Rooij et al. 2015). There is also a small body of research investigating the effects of serotonin-noradrenaline reuptake inhibitors, which indicates they can be efficacious (Mahmoudi-Gharaei et al. 2011; Park et al. 2014). Additionally, it has been suggested that at clinically relevant doses, atomoxetine acts on both the noradrenergic and serotonergic transporter (Ding et al. 2014). Selective serotoninergic drugs have been shown to reduce depression symptoms, hyperactivity and inattention in individuals with ADHD and comorbid depression (Quintana et al. 2007) and alter activity in default mode areas (Carlisi et al. 2016).

Given the prominence of 5-HT in ADHD, and the tentative data suggesting 5-HT drugs may offer effective treatment for the condition, it is unsurprising that this neurotransmitter has also been the focus of other treatment approaches, including dietary interventions. Whilst dietary modifications are not a recommended treatment for ADHD, concerns about the side effects, safety and long-term use of psychostimulant medications drive individuals and their families to explore different approaches (Rucklidge et al. 2009). 5-HT synthesis and release is known to be dependent on concentration of the precursor molecule, tryptophan, an essential amino derived from our diet (Schaechter and Wurtman 1990). Tryptophan can be found in high protein foods such as turkey, chicken, beef, pork, salmon, soybeans and pumpkin seeds (Lindseth et al. 2015) meaning that modulating tryptophan levels could be a relatively straightforward intervention. However, the exact impact of tryptophan on ADHD symptoms is unclear. The aim of this systematic review is to study the effects of tryptophan modulation on symptoms of ADHD.

## Methods

This review was conducted following the PRISMA guidelines (Moher et al. 2009), which propose a comprehensive framework for increasing rigorous reporting in systematic reviews and reducing the risk for bias. The protocol for this review and an associated meta-analysis (study number permitting) was submitted to PROSPERO (Registration number: CRD42020188649).

### Inclusion criteria

Articles included in this review were trials of interventions with modulation of tryptophan in children or adults with ADHD according to the following guidelines.

#### Types of studies and intervention

All randomised controlled trials, either single or double-blinded, with an experimental tryptophan intervention were included. Trials that combined tryptophan interventions with other lifestyle manipulations, such as dieting or exercising, were included if they provided sufficient information on the effects of tryptophan modulation alone.

#### Types of participants

This review focused on any individual with a formal diagnosis of ADHD and, therefore, included both children and adults of any age. The formal diagnosis must have been made according to either of the two main diagnostic systems: an ADHD diagnosis under the Diagnostic and Statistical Manual of Mental Disorders [DSM] or a diagnosis of hyperkinetic disorders according to the International Statistical Classification of Diseases and Related Health Problems [ICD]). The diagnosis could have been made as part of the study or prior to it. All presentation or subtypes of ADHD were included. Studies were included irrespective of medication status and comorbid diagnosis of participants.

#### Outcome measures

This review focused on the following three main outcome measures: (1) global functioning/quality of life, measured through validated scales before and after the intervention; (2) cognitive and behavioural tests which measure the core symptoms of ADHD (inattention, impulsivity and hyperactivity), before and after the intervention (i.e. attention: continuous performance tasks, such as TOVA; impulsivity: the Iowa Gambling Task, Delay Discounting Task, TOVA; hyperactivity: any objective measure of motor activity, such as actigraphy); (3) scale measures of core symptoms, including Connors, ADHD Adult Self-report Rating Scale (ASRS), Barrett Impulsivity Score or any validated ADHD core symptom scale. Additional outcomes for ADHD-related symptoms measured using standardised tests or scales before and after the intervention (i.e. aggression, working memory, reward learning) were included.

### Search strategy and information sources

Two authors conducted independent searches (LD, NP). Studies were identified by searching relevant papers on the following electronic databases: MEDLINE, EMBASE, PsychINFO, clinicaltriaregister.eu, ClinicalTrial.gov, Science Citation Index Expanded (SCI-Expanded), Conference Proceedings Citation Index – Science (CPCI-S), Web of Science. The search was not limited to a specific timeline and included all eligible studies published until July 2020, when the last search was performed. Two additional criteria adopted were as follows: (1) written in English language; (2) published in a peer-reviewed journal. Database searches were supplemented by hand searches of published systematic reviews. The PICO model for clinical questions was adopted for identifying the search categories. For population, the search terms used were (attention deficit disorder with hyperactivity[mh] OR (attention[tiab] AND deficit[tiab] AND disorder[tiab]) OR ADHD[tiab] OR AD/HD[tiab] OR (hyperkinetic[tiab] AND disorder[tiab])); for intervention, this review used tryptophan; TRP; for comparison, the search terms were ((placebo*[tiab] OR PBO[tiab] OR placebos[mh]) AND (random*[tiab] OR randomized controlled trial[pt] OR randomized controlled trials as topic[mh] or random allocation[mh]); finally, for outcomes, the search terms used were attention; impulsive*; hyperactivity; function*; quality of life.

### Study selection and data extraction

**Fig. 1:**
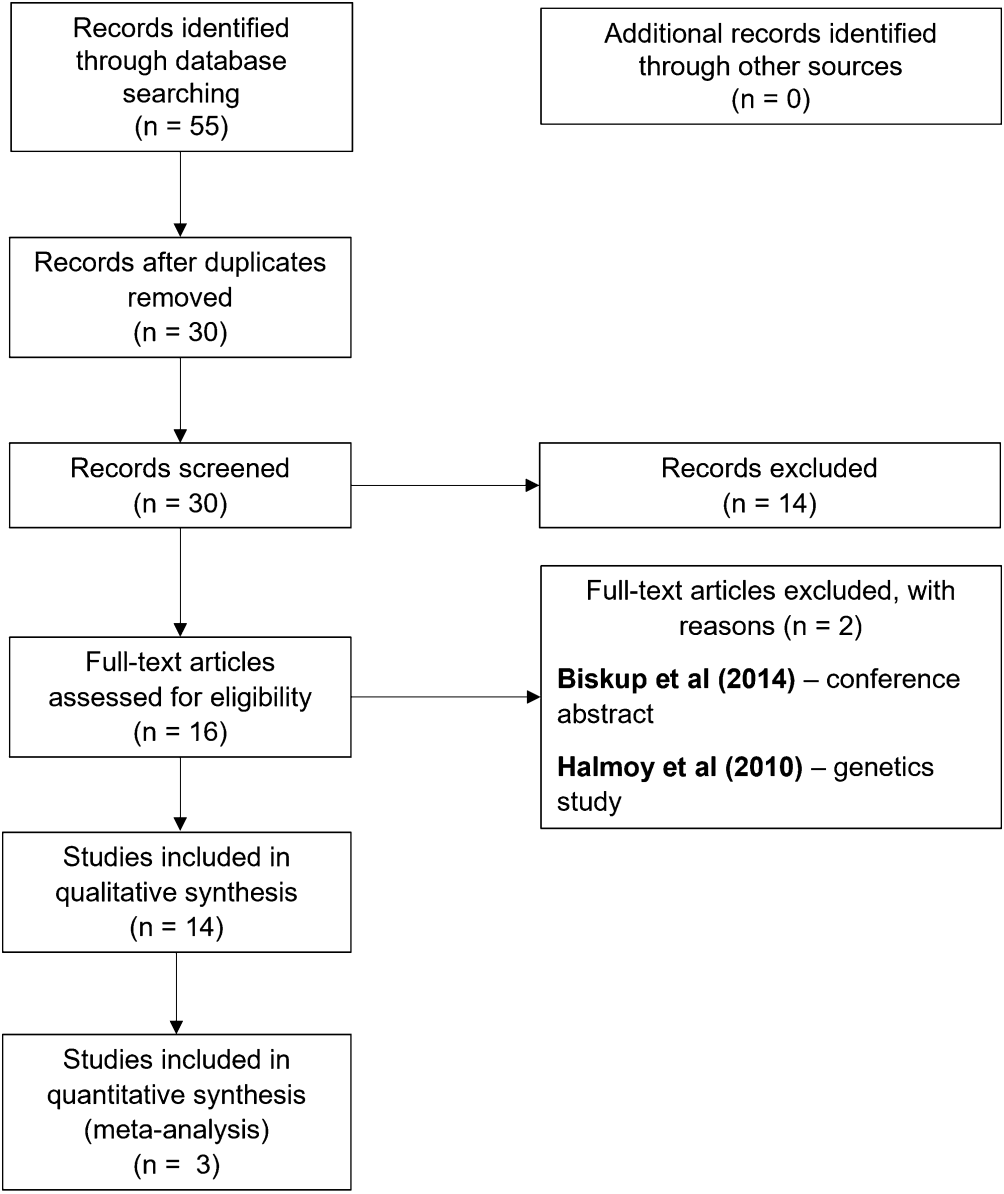
PRISMA flow diagram

The selection process was conducted by two independent authors (LD, NP) and overseen by the senior author (ED). There were no disagreements between the authors regarding the included studies. The PRISMA flow diagram (see Fig. 1) gives more information on the selection process. Different types of data were extracted from each study including the following: (1) study description and funding, (2) methodological characteristics, (3) participants’ characteristics, (4) intervention, and (5) outcome measures.

### Analysis

#### Assessment of risk of bias

The Cochrane Risk of Bias instrument for randomised controlled trials was employed to ascertain the risk of bias. Further to this, this review evaluated study quality based on sampling bias (related to sample representativeness) and measurement bias (related to inappropriate use of scales or tests to measure the pre-defined outcome).

#### Effect sizes

To measure effect, the change from baseline to study completion (objective measurement, self-report, observer rate) was used for global functioning, ADHD core symptoms and other ADHD-related symptoms. Effects sizes of the change in scores were used as the effect measure. Effect sizes were to be expressed as Hedges’ g, which accounts for sample variance and variations in sample size (Cheung et al. 2012) and the guidelines established by Cohen (1988) used for interpreting the magnitude of the effect size (0.2 = small, 0.5 = medium, and 0.8 = large).

#### Meta-analysis

A meta-analysis was planned if three or more studies using the same outcome measure were identified and fulfilled the eligibility criteria. The planned meta-analysis used standardised procedures with effects sizes (ES) being calculated for treatment–control differences for each trial and then pooled in R 4.0.5 with R Studio using the packages meta, metaphor and dmetar according to Harrer et al. (2019). If studies included more than one measurement time point, the first time point measured post-intervention would be used.

## Results

### Study selection

The search identified 55 studies through database searching, 25 of which were duplicates and were removed. The screening phase involved the examination of titles and abstracts of identified studies. This process excluded 14 studies and 16 studies were assessed for eligibility through examining the full text. From these, two studies were excluded as they did not fulfil the eligibility criteria. Fourteen studies met the pre-defined eligibility criteria and were included in the qualitative synthesis for the systematic review. It should be noted that all the studies included are from the same research group.

### Study characteristics

Detailed information about the methodological characteristics of the reviewed studies can be found in Table 1.

**Table 1:** Main study characteristics

Study	Participant characteristics			Subtypes	Comorbid conditions	Intervention	Main outcome	Other outcomes	Findings
	*N*	Age	Sex						
Stadler et al. (2007)*^†^	22	10.91 (1.77)	M	Not specified	*N* = 6, CD	ATD/BAL	Reactive aggression (PSAG)	Trait aggression + trait attention (CBCL)	Low Provocation (LP) trials: increased aggression under ATD. High provocation (HP) trials: increased aggression in both ATD and placebo conditions. No relationship with trait attention/aggression
Zepf et al. (2008a)	22	10.91 (1.77)	M	22 combined type	*N* = 6, CD	ATD/BAL	Behavioural inhibition (Go/No-go task)	Trait aggression (Buss-Perry)	High hostile participants had more inhibition errors under ATD; low hostile participants showed fewer errors under ATD
Zepf et al. (2008c)	22	10.91 (1.77)	M	Not specified	*N* = 6, CD	ATD/BAL	Reaction time (PSAG)	Trait attention + trait aggression + anxiousness/depressiveness (CBCL)	Positive correlation between delta HP and scores on CBCL-PBD scores. High scores had slower reaction time (RT) under ATD; low scores had faster RT under ATD
Zepf et al. (2008﻿b)	22	10.91 (1.77)	M	Not specified	*N* = 6, CD	ATD/BAL	Reactive aggression (PSAG)	Trait aggression (Buss-Perry) + trait impulsivity (IVE)	LP trials: no difference in reactive aggression under ATD. LP trials negatively correlated with trait impulsivity. HP trials: no difference in reactive aggression under ATD
Zepf et al. (2009a)	17	11.18 (1.81)	M	Not specified	Not specified	ATD/BAL	Mood (EWL)	Trait impulsivity (IVE)	No effect of ATD on mood ratings nor trait impulsivity
Zepf et al. (2009b)	16	11.25 (1.84)	M	Not specified	*N* = 2, CD	ATD/BAL	Heart rate (HR) (Polar S810)	Trait impulsivity (IVE)	Low impulsive group: lower HR under ATD. High impulsive group: no difference in HR under ATD/placebo
Zepf et al. (2010)	22	10.9 (1.8)	M	Not specified	*N* = 6, CD	ATD/BAL	Attention (test for attentional performance)	–	Lapses of attention were higher in the placebo group compared to ATD 120 min (T1) on. This difference does not hold at T2 (220 min) and T3 (300 min)
Zimmermann et al. (2012)*^†^	20:20	30.25 (9.37)/27.90 (6.01)	M	13 combined type, 7 inattentive	Not specified	ATD/BAL	Reactive aggression (PSAG)	Trait impulsivity (BIS)	LP trials: lower aggression under ATD in ADHD adults (opposite effect in controls). HP trials: no difference in any of the groups
Mette et al. (2013)	20:20	30.25 (9.37)/27.90 (6.01)	M	13 combined type, 7 inattentive	Not specified	ATD/BAL	Attention (modified CPT)	–	Group x condition: RT improved under ATD for the ADHD group and worsened in the HC group. More omission errors under ATD in both groups
Zepf et al. (2013)	22	10.95 (1.17)	M	Not specified	*N* = 4, CD	ATD/BAL	Verbal declarative memory (AVLT)	–	No effect of ATD on declarative memory
Kötting et al. (2013)*^†^	20	11.80 (1.88)	10 M:10F	Not specified	Not specified (but CD tolerated)	ATD/BAL	Reactive aggression (PSAG)	–	LP trials: increased aggression under ATD in female participants, no difference in male participants. HP trials: increased aggression under ATD in male participants
Grabemann et al. (2013)	20:20	30.25 (9.37)/27.90 (6.01)	M	13 combined type, 7 inattentive	Not specified	ATD/BAL	Affective prosody (Tubingen Affect Battery)	Decision time (PSAG) + trait impulsivity (IVE)	No sig effect of condition on affective prosody and no group x treatment interaction. Sig interaction between group and correct responses = > the ADHD group had fewer correct responses on the second subtask
Von Polier et al. (2014)	15	11.8 (1.9)	7 M:8F	Not specified	Not specified	ATD/BAL	Hyperactivity (electrodermal activity)	Reactive aggression (PSAG)	No condition x SCR amplitudes nor gender x condition interaction. Change in SCR under ATD neg correlated with baseline aggression
Biskup et al. (2016)^**†**^	12:10	15.5 (1.7)/16.1 (1.7)	M	Not specified	None	ATD/BAL	Default Mode Network (DMN) connectivity (rs-fMRI)	–	Differential functional connectivity between DMN and premotor cortex, somatosensory cortex under ATD in ADHD

*M*, Male, *F*, Female; *CD*, Conduct Disorder; *ATD*, Acute Tryptophan Depletion, *BAL*, Balanced; *PSAG*, point-subtraction aggression game, *EWL*, mood rating scale, *ALVT*, Auditory Verbal-Learning-Test; *CBCL*, Child Behaviour Check List, *IVE*, Eysenck’s Impulsivity Inventory/Impulsiveness and Venturesomeness Questionnaire, *BIS*, Barratt Impulsiveness Scale; *LP*, Low provocation, *RTD*, Rapid tryptophan depletion test; *HP*, High provocation, *PBD*, Pediatric bipolar disorder, *RT*, Reaction Time, *HR*, Heart rate.

Studies with * were included in the meta-analysis. The four studies bolded and marked with ^†^ are the only studies using independent samples.

### Country of data collection

The studies included in this review were all conducted in Germany.

### Participant characteristics

The reviewed studies included data from 74 unique participants with ADHD and 30 healthy controls. Since the reviewed studies were largely conducted by the same authors, some participant samples were used across multiple studies despite focusing on different outcome measures in the published articles. For instance, Mette et al. (2013) and Grabemann et al. (2013) used the same sample as Zimmermann et al. (2012), and, similarly, an identical sample as in Stadler et al. (2007) was used in Zepf et al. (2008a), Zepf et al. (2008b), Zepf et al. (2008c), Zepf et al. (2009a, b), Zepf et al. (2010), Zepf et al. (2013), Kötting et al. (2013) and Von Polier et al. (2014). Biskup at el. (2016) used a unique sample. Therefore, this review is based on four different samples and the number of participants included in this review considers that the aforementioned studies presented data from the same participants. Regarding gender distribution, most studies (12/14) recruited exclusively male participants (ADHD *N* = 64, Healthy controls *N* = 10) compared to female participants (ADHD, *N* = 10, no studies using female healthy controls). Three studies focused on adults with ADHD, aged between 19 and 46 years (Mette 2013; Zimmermann 2012; Grabemann 2013), although these three studies used the same sample (*N* = 40; ADHD = 20, HC = 20). The rest of the included studies involved children and adolescents, with ages between 9 and 17 years.

Included studies recruited healthy controls and participants with a formal diagnosis of ADHD. The presence of an ADHD diagnosis was confirmed by a psychiatrist in all studies. Participants with ADHD were recruited from outpatient and private clinics, whilst healthy controls were recruited from adjacent hospitals and universities (Mette 2013; Zimmermann 2012; Grabemann 2013; Biskup et al. 2016). Healthy controls were ineligible if they reached cut-off scores on various self-report questionnaires, including the ADHD self-report scale, the Beck Depression Inventory, the Wender Utah Rating Scale, and the Barratt Impulsiveness Scale.

Medication can be a confounder in interventional studies, and it is important to establish its effects on the participant and how long these effects last for. Six of the included studies did not specify the number of participants taking medication, despite highlighting that medication was interrupted 24 h prior to each of the study days. One study (Zimmermann et al. 2012) mentioned that methylphenidate was interrupted 2 days prior to the study days for those prescribed long-acting formulations, but no further details were given regarding the number of participants in this category. Three participants from this study were also receiving selective serotonin reuptake inhibitors (SSRIs) but stopped them several weeks prior to the study. Out of the studies that offered medication details, 79% of the ADHD participants were taking methylphenidate, a psychostimulant medication prescribed for ADHD management, although authors did not provide details on formulations. The argument for stopping medication 24 h prior to testing was that the half-life of methylphenidate is approximately 3 h (Swanson and Volkow 2002). Furthermore, none of the reviewed studies measured medication adherence, as medication nonadherence has been shown to be common in ADHD, particularly in children and adolescents who represent the majority of participants in this review (Adler and Nierenberg 2010).

Last, intelligence is relevant for understanding study instructions and cognitive assessments. Thirteen studies included a measure of intelligence quotient (IQ) as an exclusion criterion, including the Wechler Adult Intelligence Scale, the first part of the Culture Fair Test, the HAWIK-IV, HAWIK-II or the K-ABC. The most common IQ cut-off was 85, although three studies used a cut-off of 75 (Zimmermann 2012; Mette 2013; Grabemann 2013) and one did not specify the cut-off score (Zepf et al. 2013) but reported that the mean IQ of the sample was 102.64 (SD = 9.83). The only study that did not include any information regarding intelligence was von Polier et al. (2014).

### Outcome measures

#### Primary outcomes

Reactive or impulsive aggression, measured using a point-subtraction aggression game (PSAG), was the main outcome in five studies (Kötting et al. 2013; Stadler et al. 2007; Zepf et al. 2008b, c; Zimmermann et al. 2012). This type of aggression can be distinguished from instrumental aggression, where an aggressive act is carried out with the primary goal of reward, as opposed to as an impulsive reaction to provocation (Coccaro et al. 2015) Two studies evaluated attention as the main outcome, measured with a modified continuous performance task (CPT) (Mette et al. 2013) and the Test for Attentional Performance (Zepf et al. 2010). One study targeted behavioural inhibition with a CPT (Zepf et al. 2008a), one evaluated aggression by measuring electrodermal activity (von Polier et al. 2014) and another with heart rate (Zepf et al. 2009b), one focused on verbal declarative memory (AVLT) (Zepf et al. 2013), one targeted affective prosody, measured using the Tubingen Affect Battery (Grabemann et al. 2013) and finally, one measured functional connectivity in the default mode network using resting state functional magnetic resonance imaging (rs-fMRI) (Biskup et al. 2016).

#### Secondary outcomes

Four studies had trait impulsivity as a secondary outcome (Zepf et al. 2008a; Zepf et al. 2009a, b; Zimmermann et al. 2012), using the Barratt Impulsivity Scale (BIS), the Buss-Perry Impulsivity Questionnaire or the IVE, two used trait aggression as a secondary measure (Stadler et al. 2007; Zepf et al. 2008a), measured using the Child Behavioural Check List (CBCL) or the Buss-Perry Impulsivity Questionnaire, and one used trait attention again using the CBCL (Stadler et al. 2007).

### Methodological characteristics

All studies were empirical and used a double-blinded randomised crossover design. Randomised controlled trials are considered the gold standard in interventional research (Green 2006). Only one study assessed the main outcome using a self-reported scale (Zepf et al. 2009a).

#### Tryptophan intervention characteristics

Several characteristics were analysed with respect to the tryptophan intervention: namely, the method used in acute tryptophan depletion (ATD); the control arm; the administration procedure; and, finally, participants’ allocation to the study’s arms.

To experimentally induce an acute tryptophan depletion, the included studies used a physiological serotonergic challenge which temporarily reduces the availability of the essential amino acid tryptophan in the central nervous system. This procedure also increases competition for the mechanism that actively transports tryptophan into the brain and, through the administration of an amino acid mixture, stimulates protein synthesis in the liver which contributes to the reduced availability of tryptophan in the plasma and, subsequently, across the blood–brain barrier (Stewart et al. 2020). The three studies recruiting adult participants used the Moja et al. protocol (Grabemann et al. 2013; Mette et al. 2013; Zimmermann et al. 2012) while the ones focusing on children and adolescents employed the Moja-De protocol. The acute tryptophan depletion protocol developed by Moja and colleagues contains a mixture of seven amino acids, excluding tryptophan and has been shown to reduce plasma tryptophan levels reliably in adults (Moja et al. 1988). The Moja-De protocol is a modified protocol in which the seven amino acids are dosed according to individual body weight (1.32 g l-Phenylalanine, 1.32 g l-Leucine, 0.84 g l-Isoleucine, 0.5 g l-Methionine, 0.96 l-Valine, 0.6 l-Threonine and 0.96 g l-Lysine per 10 kg body weight), and the amount of methionine and amino acids are reduced compared to the original mixture (Dingerkus et al. 2012; Stewart et al. 2020). These modifications make it safe and effective in children and adolescents, reducing the side effects frequently observed in the original protocol.

All studies included in this review had a control arm (balanced/placebo condition), consisting of the same amino acid mixture used in the depletion procedure, but with added tryptophan (0.7 g per 10 kg of body weight). Although the acute tryptophan depletion procedure is temporary and reversible, some studies offered participants a multivitamin drink containing one third of the daily dose of nicotine-adenine dinucleotide, a tryptophan-derived vitamin, a soup and a protein-rich meal after the experiment.

Regarding the administration procedure, the acute tryptophan depletion and the balanced condition were administered on two separate study days in all fourteen studies, at least five to seven days apart.

### Effects of tryptophan depletion intervention on ADHD for primary outcomes

#### Reactive aggression

The findings reported by the reviewed studies were mixed and are detailed in Table 1. Briefly, three studies reported increased aggression in the low-provocation trials under the ATD procedure when reactive aggression was the main outcome (Kötting et al. 2013; Stadler et al. 2007; Zimmermann et al. 2012) and one found no difference in terms of aggression between low and high provocation trials under the experimental condition (Zepf et al. 2008b). One other study used the PSAG game as the main outcome, but they focused on reaction time instead of reactive aggression per se. They found a positive correlation between delta HP, the difference in reaction time from ATD to placebo in high provocation trials and scores on the CBCL (Zepf et al. 2008c).

#### Attention

One study measured lapses of attention at three timepoints (T1—120 min, T2—220 min, T3—300 min) and found that they were higher in the control condition compared to ATD 120 min after the drink, but this difference did not hold for the other time points (Zepf et al. 2010). The second study reported a group (ADHD and HC) by condition interaction, whereby reaction times improved under ATD for the ADHD group but worsened in the control group. Omission errors were also higher under ATD in both participant groups (Mette et al. 2013).

#### Other primary outcomes

Each of the other studies included in this review measured different outcomes. One study measured behavioural inhibition using a CPT task and trait aggression and found that high hostile participants produced more inhibition errors under ATD with the opposite pattern being identified for low hostile patients (Zepf et al. 2008a). One study measuring heart rate and trait impulsivity reported lower HR in the low impulsivity group under the ATD condition (Zepf et al. 2009b). Another study measured mood and trait impulsivity using self-report measures and found no effect of the ATD challenge on either measure (Zepf et al. 2009a). Verbal declarative memory was investigated in one study but found no effect of the ATD procedure on declarative memory (Zepf et al. 2013). Another study measured affective prosody, decision time using the PSAG game and found a significant interaction between participant group and correct responses, with the ADHD group performing worse. However, the experimental condition did not have any effect on affective prosody (Grabemann et al. 2013). Electrodermal activity and reactive aggression were investigated in one study, which did not find any interactions between condition and skin conductance amplitudes. The only finding reported in this study was that changes in skin conductance under the ATD correlated negatively with baseline aggression, but aggression was not measured under the two experimental conditions (von Polier et al. 2014). Last, one study used resting state fMRI to measure default mode network connectivity under the two conditions and reported differential connectivity between the DMN, the premotor cortex and the somatosensory cortex under the ATD challenge in the ADHD group (Biskup et al. 2016).

#### Transfer and generalisation effects

All studies included in this review involved the repeated administration of behavioural or cognitive measures under the crossover design (except for Biskup et al. 2016). Nonetheless, transfer effects were not evaluated in any of these studies. Generalisation effects are also unclear, considering the small sample sizes and the fact that all studies were conducted in Germany, largely within the same research group.

### Limitation assessments

It is important to acknowledge the limitations of the 14 studies included in this review to discuss recommendations for future research investigating the effect of dietary serotonin modulation on ADHD symptoms. Regarding the design of the studies, all studies included in this review identified limited sample size as a potential limitation of their research. In addition, ten studies included in this review did not include a control group, making it difficult to evaluate whether findings are ADHD specific. Only four studies included a control group (Biskup et al. 2016; Grabemann et al. 2013; Mette et al. 2013; Zimmermann et al. 2012), but three of those used data from the same participants. Gender differences are important to be considered, but only two studies (Kötting et al. 2013; von Polier et al. 2014) included females in their samples. Some limitations regarding outcome assessment should also be mentioned. For example, Zimmermann et al. (2012) used a reactive aggression game (PSAG) in a sample of adults, despite the measure not being validated in this age group. Another limitation that was acknowledged in one study (Kötting et al. 2013) but can be applied to all is that the time left between the acute tryptophan depletion and the behavioural tasks was presumably chosen arbitrarily. Finally, seven of the included studies permitted the inclusion of individuals with conduct disorder in their studies but due to the small sample size were unable to analyse the data from these individuals separately, introducing further heterogeneity in the studies. Similarly, across all papers there was little reference to ADHD subtypes and no analysis of this was possible due to either lack of information about subtypes or small numbers.

Additional limitations include the complexity of the serotonergic system which should be considered when interpreting data acquired in the acute tryptophan depletion condition whereby molecular imaging techniques such as PET could prove useful, and the fact that none of the studies included in this review measured plasma tryptophan levels, with the exception of one (Grabemann et al. 2013). Although all studies included in this review recruited participants with ADHD, only three of the included studies measured the core ADHD symptoms, including attention (Mette et al. 2013; Zepf et al. 2010) and motor impulsivity (Zepf et al. 2008a). Instead, the other eleven studies focused on reactive aggression, executive functions or mood, which despite being relevant in the presentation of ADHD, are not core symptoms. None of the included studies investigated all three ADHD core symptoms (attention, impulsivity and hyperactivity).

### Risk of bias

Supplementing the analysis, a summary of possible risks of bias across the studies reviewed here are presented in Table 2. In terms of the random sequence allocation, all studies included in this review were randomised, double-blinded crossover trials whereby participants completed the experimental procedure under both conditions, such that they were considered to carry low risk of bias in this respect. Furthermore, although the categorisation of participants (e.g. ADHD diagnosis) can be considered non-random, the diagnosis was confirmed in each case by a mental health professional (e.g. child psychiatrist). The risk for allocation concealment, blinding of participants and personnel and the blinding of outcome assessments were classified as unclear due to lack of information in the reviewed studies. In terms of incomplete outcome data, three studies reported dropouts and explicitly stated how they handled missing data and, as such, these were classified as low risk (Stadler et al. 2007; von Polier et al. 2014; Zepf et al. 2008a) while for the others the risk of bias in this regard remained unclear due to insufficient information. Finally, assessment of other sources of bias involved sampling bias and measurement bias. Sampling bias was rated as high in all studies included in this review due to self-selected samples, the lack of probability-sampling techniques and the recruitment of male-only samples (except for Kötting et al. 2013 and von Polier et al. 2014). Furthermore, measurement bias was high in most studies due to inconsistent conceptualisation of the outcome measures, poor measurement of the outcome measures (except for Biskup et al. 2016), including inconsistencies between the tryptophan depletion challenge and administration of the tasks, and the inconsistent selection of instruments to evaluate outcomes.

**Table 2:** Assessment of risk of bias in individual studies

Study	Selection bias		Reporting bias	Performance bias	Detection bias	Attrition bias	Other bias	
	Sequence generation	Allocation concealment					Sampling bias	Measurement bias
Stadler et al. (2007)	–	?	–	?	?	–	+	+
Zepf et al. (2008a)	–	?	–	?	?	–	+	+
Zepf et al. (2008c)	–	?	–	?	?	?	+	+
Zepf et al. (2008b)	–	?	–	?	?	?	+	+
Zepf et al. (2009b)	–	?	–	?	?	?	+	+
Zepf et al. (2009a)	–	?	–	?	?	?	+	+
Zepf et al. (2010)	–	?	–	?	?	?	+	+
Zimmermann et al. (2012)	–	?	–	?	?	?	+	+
Mette et al. (2013)	–	?	–	?	?	?	+	+
Zepf et al. (2013)	–	?	–	?	?	?	+	+
Kötting et al. (2013)	–	?	–	?	?	?	+	+
Grabemann et al. (2013)	–	?	–	?	?	?	+	+
Von Polier et al. (2014)	–	?	–	?	?	–	+	+
Biskup et al. (2016)	–	?	–	?	?	?	+	–

– low risk

+ high risk

^?^ unclear risk

### Effect sizes

Effect sizes (Hedges’ *g*) were calculated for all studies providing sufficient information for these calculations for the key outcome variables presented following RTD. Using the PSAG measure of reactive aggression, Zimmerann et al. (2012), reported a medium effect size (*g* = 0.73, SE = 0.33, 95% CI [0.08, 1.37]), as did Zepf et al. (2008b) (*g* = 0.72, SE = 0.44, 95% CI [− 0.14, 1.59]), whilst Stadler et al. (2007) had a smaller effect size for the same measure (*g* = 0.45, SE = 0.43, 95% CI [− 0.40, 1.30]), and Kötting et al. (2013) reported a greater effect, again with the same measure (*g* = 1.75, SE = 0.61, 95% CI [0.56, 2.94]). Biskup et al. (2016), using rs-fMRI reported a large effect size in the right superior premotor cortex (*g* = − 2.37, SE = 0.54, 95% CI [− 3.43, − 1.31]), Mette et al. (2013) reported a large effect size for omissions (*g* = 0.98, SE = 0.34, 95% CI [0.32, 1.63]) and reaction time (*g* = -0.82, SE = 0.33, 95% CI [− 1.46, − 0.17]), Zepf et al. (2010) reported small effect size for omissions (*g* = 0, SE = 0.43, 95% CI [-0.84, 0.84]), reaction time (*g* = 0.19, SE = 0.43, 95% CI [− 0.65, 1.02]), and commissions (*g* = − 0.15, SE = 0.43, 95% CI [− 0.99, 0.69]). Zepf et al. (2009b) reported a large effect size for heart rate in high impulsive participants (*g* = 2.20, SE = 0.45, 95% CI [1.31, 3.08]). Finally, Zepf et al. (2013) reported small effect sizes for immediate memory span (*g* = 0.21, SE = 0.43, 95% CI [− 0.63, 1.05], learning efficiency (*g* = 0.18, SE = 0.43, 95% CI [− 0.66, 1.01] and retroactive inhibition (*g* = − 0.14, SE = 0.43, 95% CI [− 0.98, 0.70], but medium effect size for recognition (*g* = 0.49, SE = 0.43, 95% CI [− 0.36, 1.34].

#### Meta-analysis

Following the pre-registered protocol, a meta-analysis was performed if three or more studies measured the same construct. As reported previously, most studies included in this review measured different constructs, with reactive aggression being the sole construct measured in three studies (see Table 1). Due to the small sample size (*k* = 3) and high heterogeneity among the included studies with regards to age and gender, the results of this meta-analysis are unlikely to be generalisable and, hence, the resulting meta-analysis is reported in the Supplementary Material for information only.

## Discussion

This review aimed to assess existing evidence for a role of tryptophan in modulating symptoms of ADHD. The findings from 14 studies provided data on a range of different measures including the core symptoms of attention and impulsivity, and several ADHD-related constructs such as aggression, mood and memory. Though the reviewed studies recruited individuals with ADHD, there was little information on the core symptoms of ADHD, namely inattention, impulsivity and hyperactivity. Attention, as measured by lapses of attention (Zepf et al. 2010), omission errors and reaction time (Mette et al. 2013), was mixed with increases and decreases associated with ATD in the different studies. The most consistently examined construct in the studies reviewed was reactive or impulsive aggression. Data here, whilst still limited, indicated that decreased tryptophan could heighten aggression. Based on this we could tentatively speculate that increased consumption of high protein foods containing tryptophan may reduce aggressive behaviour and that this warrants further attention.

It is noteworthy that despite considerable speculation on the role of serotonin and tryptophan in aggression and ADHD, data were too limited for a meaningful meta-analysis. Only three studies were suitable for inclusion and these were highly heterogeneous meaning results are unlikely to generalise beyond the current sample. For example, Kötting et al. (2013) recruited male and female participants, whilst the two other studies focused on male participants only (Stadler et al. 2007; Zimmermann et al. 2012). Regarding age, Zimmermann et al. 2012 was the only study out of the three to recruit an adult sample. Furthermore, the small number of studies overall prevents subgroup analyses (Schwarzer et al., 2015). Variation in gender and age could be particularly critical in this research for several reasons. First, there is some evidence to indicate that tryptophan levels differ in males and females irrespective of diagnostic status and that they can fluctuate across time. Hestad et al. (2017) measured tryptophan concentrations, in addition to kynurenine and the ratio between kynurenine and tryptophan, in a sample of individuals with and without depression, and found that women had lower levels of both serum and cerebrospinal fluid tryptophan compared to men, irrespective of their diagnostic status. Similarly, another study on 426 individuals with lifetime affective disorder reported significant differences between men and women regarding the changes in tryptophan, kynurenine and their ratio over a 6-week period, reporting lower serum levels in women (Reininghaus et al. 2019). Second, reactive aggression is a well-documented construct which has been shown to vary, at least overtly, between genders (e.g. Archer 2004). For example, a meta-analysis of 148 studies evaluated gender differences between direct and indirect aggression in childhood and adolescence and reported increased direct aggression in boys compared to girls, and similar levels of indirect aggression, irrespective of age, ethnicity or country (Card et al. 2008). However, a large body of research evaluates aggression using laboratory paradigms, such as the PSAG, which is based on direct retaliation. This can pe problematic because women tend to engage in more indirect forms of aggression and thus it is unclear the extent to which aggression, as measured using laboratory paradigms, is a realistic representation of female aggression (Denson et al. 2018). Though there is some debate regarding the measurement of aggression and how accurately these measurements describe female aggression, it is likely that differences in aggression exist to some extent and they should be taken into account in future work investigating tryptophan modulation. Third, there are notable differences in the presentation, or even aetiology, of ADHD according to gender, age and the age of diagnosis. It has been documented that inattentiveness (compared with impulsivity and hyperactivity) is more prominent in females with ADHD and that females may develop strategies for masking their symptoms (Quinn and Madhoo, 2014; Young et al. 2020) which could impact on laboratory testing. Moreover, the clinical presentation of ADHD in adulthood is complex and the expression of symptoms changes with age. In particular, hyperactivity symptoms have been suggested to be less important in adulthood (Martel et al. 2012) and there is increasing evidence suggesting that childhood and adult ADHD groups are non-overlapping (e.g. Moffitt et al. 2015).

Based on the complex nature of aggression, the serotonergic system and their role in ADHD, it is important that covariates such as age and gender are pulled apart. Nonetheless, the small number of studies in this review meant that meaningful subgroup analyses could not be performed. For example, Schwarzer et al. (2015) suggest, as rule of thumb, that subgroup analyses should only be conducted in cases where the sample size exceeds or equals ten studies. Consequently, in light of the current analysis, the finding that decreased tryptophan could heighten aggression should be interpreted with caution and by taking into consideration the aforementioned caveats. Nonetheless, investigating the effects of tryptophan depletion in individuals with ADHD warrants further investigation, which should factor in that tryptophan levels and their subsequent effects on cognition, mood and behaviour are likely susceptible to fluctuations based on age and gender.

Furthermore, it is important to recognise that whilst the studies provide some support for a role for tryptophan in aggression, they do not provide evidence of any specific mechanism by which tryptophan could reduce aggression. Historically, it has been assumed that tryptophan modulation exerts effects via serotonin synthesis (van Donkelaar et al. 2011). However, the association between serotonin and aggression is contentious. Whilst meta-analyses do support a relationship between the two, the effect size is small, with serotonin explaining just over 1% of the variance in aggression behaviour (e.g., Duke et al. 2013). Despite this, there is some consensus on *how* serotonin might impact aggression with various models essentially proposing that serotonin reduces reactivity to stimuli, which in turn creates decreased irritability and a greater level of behavioural constraint (Spoont 1992; Linnoila and Virkkunen 1992; Coccaro et al. 1992). Key brain structures identified are the prefrontal cortex, including the orbitofrontal cortex and anterior cingulate (Coccaro et al. 2015). Irrespective of the link between serotonin and aggression, the assumption that tryptophan exerts its effects via serotonin has also been questioned, with more recent studies offering alternative mechanisms of action of tryptophan including alternations to the brain-derived neurotrophic factor (BDNF), nitric oxide synthase, kynurenine metabolites, which can then impact on NMDA receptor function, cerebral blood flow, and microtubules (van Donkelaar et al. 2011; Yousefzadeh et al. 2020). Furthermore, research already implicates BDNF (Guyon et al 2021) and NMDA receptors (Zhang et al 2021) in aggression and BDNF, nitric oxide and kynurenine are all linked to ADHD (Molina-Carballo et al. 2021; Sharma et al. 2021; Sari et al. 2020), suggesting these mechanisms may be particularly relevant to the studies reviewed here.

Regardless of the underlying mechanism, there are several points to consider when interpreting the relationship between tryptophan and reactive aggression in ADHD based on the current analysis. First, the inclusion of participants with comorbid conduct disorder makes it hard to establish if these effects are specific to ADHD. Second, the reviewed studies used relatively small samples which have been shown to produce effect sizes roughly twice as large compared with large-scale studies (Cheung and Slavin 2016). Third, despite using RCT designs, participants completed the same set of measures over two study days in a crossover approach, which could have inflated the effect sizes as the two measurements are likely to be dependent (Bakker et al. 2019; Cuijpers et al. 2017). Fourth, as mentioned in the results, the inclusion of only a small number of studies in the meta-analysis means that publication bias cannot be completely ruled out.

The studies reviewed do have several strengths. Firstly, they all used a robust experimental design, most typically a double-blind randomised crossover design. Second, in all cases diagnosis of ADHD was confirmed by psychiatrists and healthy controls who provided ratings which could indicate undiagnosed ADHD were excluded. Additionally, the measures used were largely objective and validated instruments, with minimal reliance on self or parent-report measures. Despite these strengths there are some important limitations to the studies included. The majority of the 14 studies focused on child populations and male participants, with only three studies considering adults with the disorder and two including female participants. Additionally, all studies were based in Germany and conducted by the same research group. There was also a lack of clarity over medication type and dose as well as adherence. All studies utilised ATD meaning the focus was on reducing tryptophan levels rather than increasing them. This approach means that floor effects are possible, if individuals already have low levels, as is possible for those showing high impulsivity (Oades 2008; Pattij and Vanderschuren 2008). An approach that incorporated tryptophan depletion and loading may be stronger and has been used to examine other conditions (Lieben et al. 2018).

To our knowledge this is the first systematic review to examine the effects of tryptophan modulation in ADHD. However, it is important to acknowledge we may have missed some eligible studies because the search strategy was limited to a selection of databases. Coding of articles was also subjective, although each article was coded independently by two authors and all authors agreed on final coding. Another limitation of the current study was the small number of studies and participants included with only 14 studies incorporated and less than 100 participants. In addition, the studies included all stemmed from the same research group, which may mean the studies would not generalise beyond that. Furthermore, meta-analysis and assessment of publication bias was only possible on the measures of aggression, and the results should be interpreted conservatively considering the previously discussed caveats.

### Implications for clinical practice and research

The limitations of current medications for ADHD have served to drive individuals with the condition and their families to explore different approaches (Rucklidge et al. 2009), including dietary modulation. The evidence reviewed here suggests that there is insufficient research supporting tryptophan modulation as impacting the core symptoms of ADHD. There is slightly stronger evidence to suggest that tryptophan modulation may impact on aggressive behaviours, such that we can hypothesize increases in tryptophan would decrease aggression, but this research is still in its early stages and confounded by many factors (e.g. gender, age, age of ADHD diagnosis etc.) meaning such tryptophan modulation approaches should not be recommended for people with ADHD at this stage.

Future research in this area needs to address the shortcomings of the identified studies. For example, it is critical that these small-scale studies are now followed-up with larger studies, providing sufficient statistical power and a suitable control arm. These studies should also be conducted across a wider spread of geographical locations because the context and regulation of dietary supplementation is known to vary by country (Thakkar et al. 2020). Studies should also include both genders. This is particularly important for adult population where the gender bias towards boys, which has often been reported in children, is thought to be diminished or even reversed, meaning females must be considered equally (Breda et al. 2021). In line with this, it is also critical that adults are better represented in such research going forward. The prevalence of ADHD in adults and children is similar (Polanczyk et al. 2015; Simon et al. 2009; Zalsman and Shilton 2016) and there are fewer non-drug treatment options available to adults (NICE 2019), meaning there is arguably a greater need for alternative treatments to be investigated in this cohort. Furthermore, within both child and adult populations, future studies should try to distinguish according to key clinical features presentation type and common comorbidities because these have been shown to impact on treatment outcomes (Reale et al. 2017). Further consideration of current treatment may also be beneficial. Additionally, given that all studies employed a tryptophan depletion paradigm, future studies should consider alternative approaches such as tryptophan loading. Finally, at present, most studies did not measure core ADHD symptoms or key factors in functional impairment such as social skills and therefore, future studies should consider utilising objective, validated measures of these symptoms alongside methods which could help disentangle the molecular mechanisms by which the acute tryptophan depletion challenge acts. The latter will likely need to draw on pre-clinical studies.

In conclusion, the research reviewed here examining the impact of tryptophan modulation on ADHD is limited. There is some indication to suggest that altering tryptophan levels may alter aggression in some individuals with ADHD, but high sample heterogeneity, comorbidities and small samples sizes limit conclusions. Furthermore, it is critical that future studies focus on the core symptoms of ADHD and that they include adults and females, who are currently under-represented in this research.
